# Phylogenetic congruence and ecological coherence in terrestrial Thaumarchaeota

**DOI:** 10.1038/ismej.2015.101

**Published:** 2015-07-03

**Authors:** Eduard Vico Oton, Christopher Quince, Graeme W Nicol, James I Prosser, Cécile Gubry-Rangin

**Affiliations:** 1 Institute of Biological and Environmental Sciences, University of Aberdeen, Cruickshank Building, Aberdeen, UK; 2 School of Life Sciences, Centre for Biomolecular Sciences, University of Nottingham, Nottingham, UK; 3 Warwick Medical School, University of Warwick, Warwick, UK; 4 Laboratoire Ampère UMR CNRS 5005, École Centrale de Lyon, Université de Lyon, Ecully CEDEX, France

## Abstract

Thaumarchaeota form a ubiquitously distributed archaeal phylum, comprising both the ammonia-oxidising archaea (AOA) and other archaeal groups in which ammonia oxidation has not been demonstrated (including Group 1.1c and Group 1.3). The ecology of AOA in terrestrial environments has been extensively studied using either a functional gene, encoding ammonia monooxygenase subunit A (*amoA*) or 16S ribosomal RNA (rRNA) genes, which show phylogenetic coherence with respect to soil pH. To test phylogenetic congruence between these two markers and to determine ecological coherence in all Thaumarchaeota, we performed high-throughput sequencing of 16S rRNA and *amoA* genes in 46 UK soils presenting 29 available contextual soil characteristics. Adaptation to pH and organic matter content reflected strong ecological coherence at various levels of taxonomic resolution for Thaumarchaeota (AOA and non-AOA), whereas nitrogen, total mineralisable nitrogen and zinc concentration were also important factors associated with AOA thaumarchaeotal community distribution. Other significant associations with environmental factors were also detected for *amoA* and 16S rRNA genes, reflecting different diversity characteristics between these two markers. Nonetheless, there was significant statistical congruence between the markers at fine phylogenetic resolution, supporting the hypothesis of low horizontal gene transfer between Thaumarchaeota. Group 1.1c Thaumarchaeota were also widely distributed, with two clusters predominating, particularly in environments with higher moisture content and organic matter, whereas a similar ecological pattern was observed for Group 1.3 Thaumarchaeota. The ecological and phylogenetic congruence identified is fundamental to understand better the life strategies, evolutionary history and ecosystem function of the Thaumarchaeota.

## Introduction

The ecology of organisms belonging to the phylum Thaumarchaeota has been studied extensively for over 20 years since sequences associated with this lineage (then termed ‘Group 1' or non-thermophilic Crenarchaeota) were first discovered in marine waters, followed by terrestrial and other aquatic habitats (DeLong, 1998). The initial studies, focusing almost exclusively on 16S ribosomal RNA (rRNA) gene surveys, indicated a level of ecological coherence within the ‘Group 1' phylum, with some lineages dominating particular habitats (Nicol and Schleper, 2006). Since the discovery and cultivation of ammonia-oxidising archaea (AOA) (Könneke *et al.*, 2005), many of these lineages have been found to represent organisms involved in nitrogen cycling, with genome analysis demonstrating that they belonged to a distinct phylum, the Thaumarchaeota (Brochier-Armanet *et al.,* 2008). In terrestrial ecosystems, AOA often outnumber ammonia-oxidising bacteria (Leininger *et al.*, 2006; Stempfhuber *et al.*, 2014), and they dominate ammonia oxidation in a wide range of soils (Offre *et al.*, 2009; Gubry-Rangin *et al.*, 2010; Stopnisek *et al.*, 2010; Lu *et al.*, 2012; Prosser and Nicol, 2012; Alves *et al.*, 2013; Daebeler *et al.*, 2014).

The key enzyme for ammonia oxidation is ammonia monooxygenase, whose three subunits, A, B and C, are encoded by *amoA, amoB* and *amoC* genes, respectively (Sayavedra-Soto and Arp, 2011; Urakawa *et al.*, 2011). The *amoA* gene has been used extensively as a marker for both AOA and ammonia-oxidising bacteria in environmental studies. Currently, thaumarchaeotal *amoA* sequences deposited in GenBank are approximately fourfold more abundant than thaumarchaeotal 16S rRNA genes, probably reflecting the more frequent use of *amoA* as a marker gene. However, although ammonia oxidation has been demonstrated in the 16S rRNA-defined lineages Group 1.1a, 1.1a-associated, 1.1b and those from thermophilic *Nitrosocaldus* lineage (representing all known AOA), very little is known about the other thaumarchaeotal groups represented only by 16S rRNA gene sequences (for example, Groups 1.1c, 1.3) (Pester *et al.*, 2011). Indeed, these lineages remain largely uncharacterised with no pure culture or enrichment described. No clear ecological function can be attributed to these groups, although one study (Weber *et al.*, 2015) could find no evidence of a role for Group 1.1c Thaumarchaeota in ammonia oxidation. Thaumarchaeotal *amoA* and 16S rRNA genes therefore do not represent the same phylogenetic diversity and no in-depth comparison of the diversity recovered by these two genes has been performed. Previous study (Nicol *et al.*, 2008) suggests congruence between 16S rRNA and *amoA* gene phylogenies for the AOA with similar tree topologies at high taxonomic resolution. No comparison of these phylogenies is currently available at a finer taxonomic scale, closer to the traditional genus or species levels, preventing comparison of findings from studies using different markers.

Environmental analyses (Nicol *et al.*, 2008; Gubry-Rangin *et al.,* 2011; Pester *et al.*, 2011) and microcosm studies (Nicol *et al.*, 2008) using the archaeal *amoA* gene provide strong evidence that pH is a key factor driving niche specialisation of AOA. Detailed phylogenetic analysis indicates ecological coherence with respect to soil pH (Gubry-Rangin *et al.*, 2011) and some lineages can be described as being adapted to specific pH ranges (that is, acidophile, neutrophile or alkalinophile lineages). Ecological coherence of a given lineage implies that its members share life strategies or specific traits that distinguish them from other taxa (Philippot *et al.*, 2010). Other soil characteristics will influence AOA community composition (Prosser and Nicol, 2012; Zhalnina *et al.*, 2012). For example, analysis of archaeal 16S rRNA genes indicated that C:N ratio is associated with thaumarchaeotal distribution (Bates *et al.*, 2011) and correlations between thaumarchaeotal community composition and other factors have also been found, including water content (Stres *et al.*, 2008; Angel *et al.*, 2010; Richter *et al.,* 2014), total nitrogen content (Pester *et al.*, 2012), organic carbon content (Pester *et al.*, 2012; Bates *et al.*, 2011), temperature (Bates *et al.*, 2011; Stres *et al.*, 2008; Tourna *et al.*, 2008), oxygen concentration (Szukics *et al.*, 2010) and concentrations of several heavy metals (Cao *et al.*, 2011). However, none of these studies involves thorough analysis of the relationship between community structure and environmental factors over a range of taxonomic scales for both *amoA* and 16S rRNA genes, potentially leading to discrepancies between studies. Indeed, we hypothesise that physicochemical factors will impact differently on the environmental distribution of Thaumarchaeota at different phylogenetic resolutions, and that these differences will reflect the evolutionary time at which specific factors started to influence microbial distribution.

We therefore hypothesise that significant congruence exists between the phylogenies of thaumarchaeotal 16S rRNA and *amoA* genes, that the environmental factors driving thaumarchaeotal distribution in soil vary at different levels of phylogenetic resolution and that pH is a major driver for all Thaumarchaeota, and not just those for which ammonia oxidation has been confirmed. We also ask whether phylogenetic congruence between 16S rRNA and *amoA* genes is significant at a finer taxonomic resolution than previously used (Nicol *et al.*, 2008). To address these aims, we sequenced and analysed 16S rRNA and *amoA* genes in 46 UK soils that have previously been shown to represent the same level of diversity as the global thaumarchaeotal diversity (Gubry-Rangin *et al.*, 2011) and for which significant contextual data (29 environmental factors) are available.

## Materials and methods

### Data collection and high-throughput sequencing

Thaumarchaeotal 16S rRNA and *amoA* gene sequences were obtained from 39 soils representing different ecosystems (agricultural, forest, moorland and grassland) over a pH range of 3.5–8.5 and these soils were a subset of 1000 soil samples taken as part of the UK Countryside Survey (http://www.countrysidesurvey.org.uk/). Seven additional soil samples were collected from a pH gradient of 4.5–7.5 from long-term experimental field plots (SRUC, Craibstone Scotland (grid reference NJ872104)). Twenty-nine physicochemical parameters were available for the 39 Countryside Survey samples and eight were available for the Craibstone soils, all measurements being performed using the same methodology (Supplementary Table S1). Soils were chosen to maximise the distribution of the available environmental factors. DNA extraction (Griffith *et al.*, 2000) and 454 high-throughput sequencing were performed for both *amoA* and 16S rRNA genes following the methodology previously described (Gubry-Rangin *et al.,* 2011) using primers CrenamoA23f and CrenamoA616r (Tourna *et al.*, 2008) for *amoA* gene and A109f (Grosskopf *et al.*, 1998) and 752r (reverse complement of 771 f (Ochsenreiter *et al.*, 2003)) for 16S rRNA (see Supplementary Information Material and Methods).

### Multivariate statistical analysis

For the 39 Countryside Survey soils, Operational Taxonomic Unit clustering of the sequences was performed at 100, 97, 95, 90, 85, 80 and 70% using the UCLUST algorithm (Edgar 2010) after ordering the sequences by abundance within each data set. These Operational Taxonomic Unit levels were chosen arbitrarily to test for the existence of thaumarchaeotal ecological coherence across a gradient of taxonomical scale. Canonical Correspondence Analysis and analysis of variance tests were performed on the relative abundance matrices (indicating the relative percentage of each cluster per soil) on each gene and taxonomic scale using the vegan package (Oksanen *et al.,* 2013) in R V3.1.0 to identify which environmental factors had a statistically significant association with community composition.

### Bayesian phylogenetic analyses and environmental specialisation

*amoA* and 16S rRNA gene sequences detected as recombinant using RDP4 software (Martin *et al.,* 2010) were removed before subsequent phylogenetic analysis. Twenty-two thaumarchaeotal 16S rRNA and *amoA* gene sequences from cultured organisms or genomic fragments (Supplementary Table S2) were also included in the alignment to facilitate further congruence analysis between the two genes. Phylogenetic analyses were performed using a Bayesian Markov Chain Monte Carlo approach (MCMC) using BEAST software package V1.8.0 (Drummond *et al.* 2012) (see Supplementary Information Material and Methods).

Group affiliation for *amoA* and 16S rRNA gene sequences was performed using the inserted reference sequences but the lack of culture representatives of several 16S rRNA-defined groups required the use of a BLAST Search. Phylogenetic cluster delineation was performed as described previously (Gubry-Rangin *et al.*, 2011), that is, a cluster of sequences was defined if all sequences displayed a sequence similarity >90% and if the root of the cluster was supported by high phylogenetic support (>70% posterior value in most cases). *amoA* gene sequences were affiliated to different clusters by correspondence to a previous *amoA* database (Gubry-Rangin *et al.*, 2011), whereas the description of 16S rRNA gene clusters was defined in this study. Abundance of each *amoA* or 16S rRNA gene cluster was estimated and representation of the relative abundance of the most abundant clusters (>5% of the total number of reads) over the pH gradient (and its best-fitting model) in the soils was performed using packages Vegan (Oksanen *et al.,* 2013) and mgcv (Wood, 2008) in R. Reconstruction of several ancestral phenotypic states was performed when environmental factors were found to influence the thaumarchaeotal community at several taxonomic resolutions in the multivariate statistical analysis and if contextual data were available in all soils (including Craibstone soils, owing to their presence in the phylogenetic tree). This ancestral state reconstruction was performed using the package Phytools (Revell and Graham Reynolds, 2012) in R assuming a Brownian motion model of evolution.

### Comparative phylogenetic analysis

The phylogenetic comparison used to assess congruence between the two genetic markers was based on 85% and 90% for *amoA* and 16S rRNA gene, respectively. These identity levels were not chosen to correspond to particular taxonomic levels, as reliable and consistent delineations of bacterial and archaeal species are not available (Doolittle and Zhaxybayeva, 2013). Rather, they were chosen to facilitate congruence analysis in generating similar numbers of pH-specialised clusters. This congruence between *amoA* and 16 S rRNA genes was assessed using several criteria. Although the inclusion of reference sequences for both genes ascertains correspondence between several clusters of the two phylogenies, the remaining relationships were mostly based on hypothetical correspondence, suggested from phylogenetic ordination, relative abundance of the reads per cluster and mean pH preference of each cluster. Statistical congruence between *amoA* and 16S rRNA genes was further performed using the Parafit function from the ape R package (Paradis *et al.,* 2004). Parafit was originally developed to test coevolution events between parasites and their hosts (Legendre *et al.,* 2002), providing a useful tool to compare similarity between phylogenies inferred from two different gene markers. Distance matrices were created for each tree using the permutation test script (Hommola *et al.,* 2009) and a relationship matrix between the two genes was manually created based on the suggested correspondence between the two phylogenetic markers. The Parafit global test was run with 999 permutations.

## Results

### Thaumarchaeotal community

After cleaning of the reads, 639 055 16S rRNA gene sequences of varying length (range 576–612 bp) and 428 129 archaeal *amoA* gene sequences of 592 bp were retrieved, corresponding to an average of 13 893 (s.d. 7603) and 10 442 (s.d. 9363) reads per sample for 16S rRNA gene and *amoA* gene, respectively (Supplementary Figure S1). Samples were not normalised by rarefaction to maximise genetic diversity of the data sets, as recommended for community comparison (McMurdie and Holmes, 2014). Only three unique sequences were detected in each data set as potential recombinants and these were removed from the phylogenetic analysis (data not shown).

The majority of 16S rRNA gene sequences corresponded to thaumarchaeotal sequences (99.8%), whereas the remainder were affiliated to the Euryarchaeota phylum. Reads corresponded mainly to ammonia-oxidiser Thaumarchaeota (84%) with 69.7%, 14.3% and 0.02% corresponding to Group 1.1b, Group 1.1a-associated and Group 1.1a, respectively, whereas 14.1% and 1.7% were affiliated to Group 1.1c and Group 1.3, respectively. Ammonia-oxidising thaumarchaeotal sequences were amplified in 44 soils, whereas Group 1.1c and Group 1.3 sequences were detected in 24 and 17 soils, respectively.

**Figure 1:**
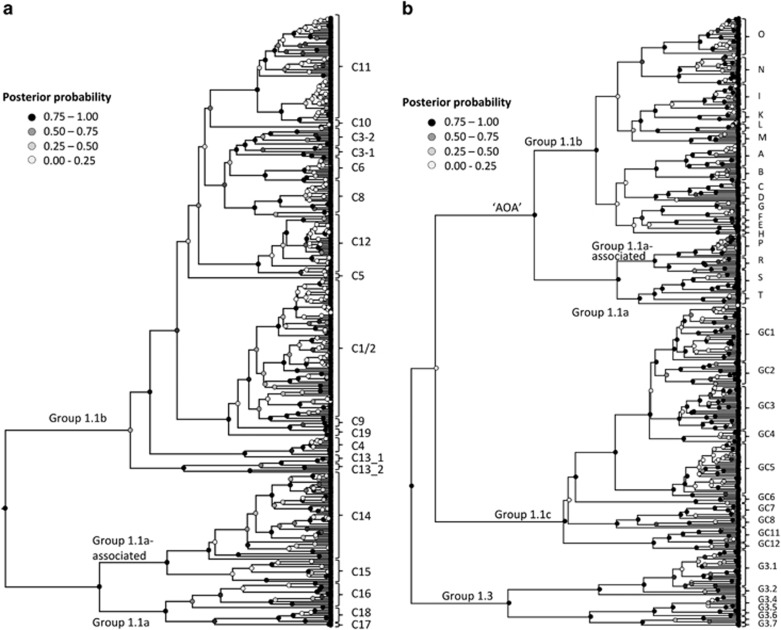
Bayesian phylogenetic trees of thaumarchaeotal *amoA* (**a**) and 16 S rRNA genes (**b**). In both trees, circles are represented for each node and the shading relates to the node posterior probability (with more confidence in the node being attributed to the darkest colour). The assignment of sequences into the different clusters is indicated near the tips of the trees.

After dereplication at 100% identity, 334 *amoA* and 408 16S rRNA gene sequences were used for Bayesian phylogenetic analysis in addition to the 22 AOA reference sequences (Supplementary Table S2). Three Thaumarchaeota groups and 19 clusters were defined in the *amoA* gene tree (Figure 1a) and five Thaumarchaeota groups and 38 clusters could be identified by the 16S rRNA gene tree (Figure 1b). Several phylogenetic clusters were abundant (that is, including >5% of the total number of reads): clusters K, I, P, O, L, A, G, P, GC1 and GC5 for the 16S rRNA gene tree (Supplementary Figure S2) and clusters C1/2, C14, C11 and C12 for the *amoA* gene tree (Supplementary Figure S3).

### Associations with environmental characteristics

Associations of environmental factors with thaumarchaeotal distribution were tested statistically after omission of seven of the 46 soils in three data sets analysed: *amoA* genes, all thaumarchaeotal 16S rRNA genes and AOA 16S rRNA genes (that is, excluding those from lineages not thought to represent AOA). For both genes, pH was associated significantly at nearly all taxonomic scales (Table 1), as found previously when *amoA* gene analysis was restricted to 85% identity (Gubry-Rangin *et al.*, 2011). However, the use of other levels of taxonomic resolution for the *amoA* gene revealed significant associations of several additional factors with the ammonia oxidiser thaumarchaeotal distribution in soils, including organic matter content, nitrogen content (mineralisable or not) and zinc and aluminium concentrations (Table 1). Interestingly, analysis of the AOA 16S rRNA gene data set also showed associations with all of these factors, except aluminium concentration, at different taxonomic resolutions (Table 1). In addition, bulk density, water content, ecosystem type and arsenic concentration explained a proportion of the thaumarchaeotal distribution (Table 1). Finally, more environmental factors influenced the global thaumarchaeotal community (based on all thaumarchaeotal 16S rRNA genes) across a wider range of taxonomic resolution with a strong influence of pH, organic matter content, nitrogen content (mineralisable or not), C:N ratio, proportion of nitrate in the mineralisable nitrogen stock, water content, ecosystem type and zinc concentration (Table 1). Spearman rank-order correlation matrices between the different environmental factors indicated that most were correlated with pH for the 16 S rRNA data set, whereas only the zinc concentration was correlated with pH for the *amoA* gene data set (Supplementary Table S3). Estimation of the mean pH preference of the most abundant clusters defined by the *amoA* gene confirmed pH specialisation for the acidophilic clusters *Nitrosotalea* C14 and *Nitrososphaera* C11 and neutro-alkalinophilic clusters *Nitrososphaera* C1/2 and C12 (Supplementary Figure S3) (Gubry-Rangin *et al.,* 2011). Distribution of the most abundant 16S rRNA-defined clusters along the pH gradient indicated five neutro-alkalinophilic clusters (*Nitrososphaera* K, L, I, O and G) and four acidophilic clusters (*Nitrosotalea* P, *Nitrososphaera* A, Group 1.1c GC1 and Group 1.1c GC5) (Supplementary Figure S4). Furthermore, all Group 1.1c and Group 1.3 clusters were acidophilic, the majority being extreme acidophiles (Supplementary Figure S2). In addition to the pH preference of these clusters, multivariate statistical analysis was used to infer mean preferences for two additional environmental factors (organic matter and water contents) that were available for all soils. This indicated that AOA are generally associated with lower soil water and organic matter content than Group 1.1c and Group 1.3 (Supplementary Figure S2), both of which were detected most frequently in forest ecosystems.

**Figure 2:**
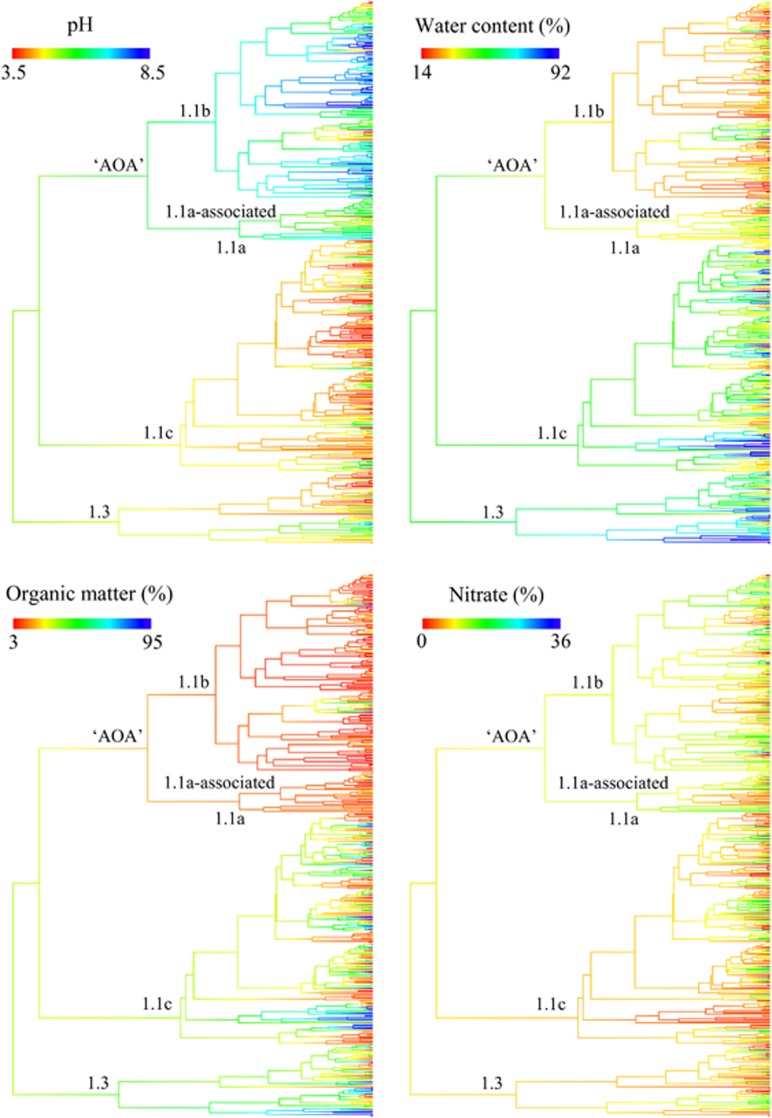
Reconstruction of the preferences of four different environmental factors along the 16 S rRNA gene phylogenetic tree of Thaumarchaeota. Reconstruction of ancestral preference for pH, water content (%), organic matter (dry weight %) and proportion (%) of nitrate in the mineralisable nitrogen pool was performed after removal of the phylogenetic tips representing culture sequences.

Ancestral reconstruction of several habitat traits was performed using the environmental factor preference of extant Thaumarchaeota (estimated by the means for each phylogenetic tip) (Figure 2). A striking difference was observed between AOA and Group 1.1c/Group 1.3 for the four habitat traits (pH, water content, organic matter content and proportion of nitrate in the mineralisable nitrogen stock, thereafter abbreviated as ‘nitrate'). Indeed, the majority of soil AOA are generally associated with neutral or alkaline soil, low water and organic matter contents and relatively high nitrate, whereas both Groups 1.1c and 1.3 are associated with the opposite characteristics. This suggests that the major changes between these three groups (AOA, Group 1.1c and Group 1.3) occurred before their diversification implying ancient environmental specialisation (Figure 2). Finally, the thaumarchaeotal ancestor was probably living in a neutral environment with high water and organic matter contents but low nitrate (Figure 2).

### Congruence analysis

*amoA* and 16S rRNA gene sequences from all available cultures or genomic fragments were included in this study to assess congruence between the two marker genes in ammonia-oxidising Thaumarchaeota. This phylogenetic comparison was based on a single resolution corresponding to 85% and 90% in *amoA* and 16S rRNA phylogeny, respectively. At this resolution, both phylogenies presented similar numbers of clusters (18 and 19, Table 1) and pH was the only common environmental factor explaining community structure. This permitted the use of two levels of correspondence between the clusters of the two phylogenies: (i) a high level of confidence when correspondence was based on the presence of one or several genomic fragments or cultures and (ii) suggested congruence between the two genes due to similar relative abundance of reads and similar pH specialisation.

**Figure 3:**
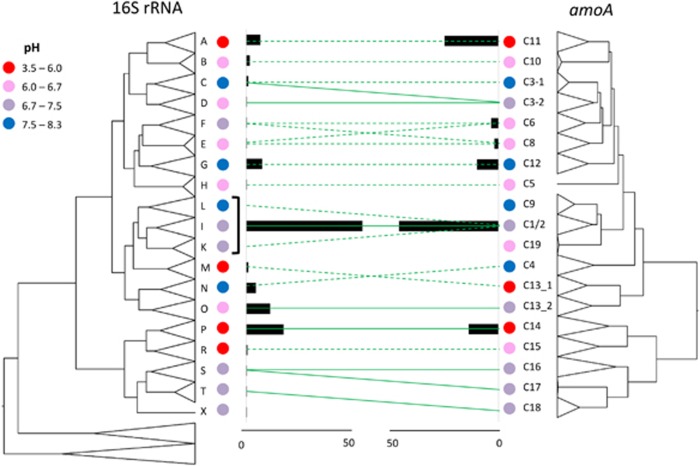
Congruence between 16S rRNA and *amoA* gene cladograms for AOA organisms. The correspondence between clusters of the two phylogenies is represented by either a solid line when a high level of confidence is attained based on the presence of one or several genomic fragments or cultures or by a dashed line when the congruence between the two genes is suggested because of similar relative abundance of reads (represented by the black bars next to the tips of each phylogeny) and similar pH specialisation (represented by the coloured circles).

This analysis generated seven *amoA* gene clusters affiliated with high confidence to seven 16S rRNA gene clusters (Figure 3). The congruence of all abundant clusters (>5% of the total number of reads) was predicted by the pH specialisation of the clusters and their relative abundances (Figure 3). The relative abundance of acidophilic clusters A and P in the 16S rRNA tree corresponded to those of C11 and C14 in the *amoA* gene tree, respectively. Grouping of the three neutrophilic–alkalinophilic 16S rRNA clusters I, K and L probably corresponded to *amoA* cluster C1/2, whereas 16S rRNA cluster G corresponded to *amoA* cluster C12. Several clusters in both phylogenies could not be associated with those in the other phylogeny, including 16S rRNA cluster X or *amoA* cluster C19, probably through difficulty in detecting rare clusters, but also potentially through primer bias. A Parafit test was applied, assuming correct affiliation between clusters, to assess incongruence between *amoA* and 16S rRNA gene phylogenies and generated a global value of 1241.34 (*P*=0.001), providing evidence of significant congruence.

## Discussion

### Ecological coherence

This study aimed to assess ecological coherence in thaumarchaeotal communities by analysing the phylogenetic association of two widely used marker genes, *amoA* and 16S rRNA, with different physicochemical properties found in a variety of temperate soils. This approach involves four major caveats that may influence the results. First, the influence of some factors may be limited by the narrow range found in the ecosystems investigated. For example, heavy metal concentrations were biased towards lower values, as only unpolluted sites were studied. The mean annual temperature range is also restricted to that within the United Kingdom, but this satisfied our aim of studying the distribution of Thaumarchaeota in natural temperate environments. Second, the approach is restricted to the analysis of correlation and therefore only allows indirect inference on which factors are driving or impacting on community composition. Nevertheless, as we analysed data sets from a contrasting range of soil types and over a range of taxonomic resolutions, we believe that using only those factors that were significant across several phylogenetic scales should allow confidence in the interpretation of correlation-based results. Third, this analysis assumes spatial and temporal homogeneity both for the microbial community and the environmental factors measured, whereas ammonia-oxidising communities and certain environmental factors such as nitrate or water content are likely to vary over the time scales required for community change (Placella *et al.*, 2012; Pereira *et al.*, 2012). However, the analysis of microbial occurrence rather than microbial activity under particular environmental conditions on such a relatively large number of samples investigated reduces this bias. Fourth, assessment of the causative nature of correlations and associations requires information on ecophysiological characteristics of different phylotypes and/or experimental studies. Such information is scarce for Thaumarchaeota, due to lack of laboratory cultures and physiological studies, and of experimental studies investigating specific factors. In this respect, the current study presents a framework for future experimental work. In addition, this approach assumes the concept of niche specialisation and differentiation rather than neutral theory.

With these caveats in mind, this study confirmed the important role of pH in thaumarchaeotal niche specialisation and analysis of both marker genes provided strong evidence of pH as a key factor, explaining community composition across the full range of taxonomic scales tested (Table 1). The data suggest that pH had an important role from a very early stage in the evolutionary history of Thaumarchaeota, whereas its influence on the distribution of extant organisms remains strong. pH adaptation reflects strong ecological coherence at various levels of taxonomic resolution, as the different groups, clusters or sub-clusters of Thaumarchaeota are adapted to specific life strategies, depending on environmental pH. Although strong ecological coherence for pH was observed with both genes (85% similarity for *amoA* and 90% similarity for 16S rRNA), the variance of pH adaptation differs between clusters with specialisation of certain clusters being more constrained than others, presenting a narrower range of conditions and thus having greater coherence. The existence of non-specialised clusters (previously assigned as generalists, Gubry-Rangin *et al.,* 2011) suggests that the ecological coherence of the Thaumarchaeota phylum is lineage-dependent, as frequently observed in other organisms (Philippot *et al.,* 2010; Koeppel and Wu 2012; Gobet *et al.,* 2014).

### AOA: marker-dependent community distribution?

In addition to confirmation of the strong effect of pH on AOA, this study suggests that other environmental factors are also driving thaumarchaeotal community distribution, especially at taxonomic resolutions other than previously performed (Gubry-Rangin *et al.*, 2011). It must be noted that temperature was not detected as significant in the study but we believe that it has contributed to ancestral microbial distribution, at least before differentiation of the known aerobic and mesophilic ammonia-oxidising Thaumarchaeota (Groups 1.1b, 1.1a and 1.1a-associated) (Groussin and Gouy, 2011). Although pH had the strongest association with ammonia-oxidising thaumarchaeotal community composition, multiple environmental factors influenced distribution patterns of both genes at several taxonomic resolutions: organic matter, nitrogen, total mineralisable nitrogen and zinc concentration. Covariance of organic matter with many types of soil microbial activity makes the clear link between organic matter and thaumarchaeotal community difficult to interpret, but may relate to mixotrophic growth, which has been shown in some cultivated Thaumarchaeota (Lehtovirta-Morley *et al.*, 2014; Tourna *et al.*, 2011; Stahl and de la Torre, 2012). However, the influence of mixotrophy on ammonia oxidiser ecology in natural habitats is not currently well understood and there has been little investigation of its role in the distribution of Thaumarchaeota in soil (Prosser and Nicol, 2012). The detection of significant associations with nitrogen concentrations and total mineralisable nitrogen content in the soil are consistent with their role in utilising ammonia produced through mineralisation. Finally, the influence of zinc concentration on AOA was also detected and its effect on the activity and abundance of AOA was previously demonstrated (Mertens *et al.*, 2009). The fact that Zn is the only metal showing significant correlation with pH (Supplementary Table S3) supports the hypothesis that archaeal nitrification activity recovery after Zn contamination may depend on the AOA community present.

Surprisingly, the analyses performed on the two genes did not indicate significant associations with the same environmental factors, even when comparing different taxonomic resolutions between the *amoA* and 16S rRNA genes. This can be explained by differences in the diversity of the two genes resulting from different evolutionary rates and to the strong selection pressures on *amoA* (Biller *et al.*, 2012). In addition, the 16S rRNA gene is considered to be a component of the thaumarchaeotal core genome, with clearly a more essential role in cell functioning than *amoA*. This predicts a higher level of diversity for *amoA* than for 16S rRNA genes, which is indicated by simplistic comparison of the number of clusters and higher percentage of similarity based on the richness measured for 16S rRNA genes (Table 1). Based on the 90% identity for 16S rRNA gene, roughly equivalent to 85% similarity for *amoA* in term of richness, it is striking that analysis of *amoA* genes revealed association with fewer environmental factors than the 16S rRNA data set, as only pH was previously detected (Gubry-Rangin *et al.*, 2011) and the addition of many environmental factors did not change this results. This may be due to differences in ‘detection limits' but an additional hypothesis is that 16S rRNA is more likely to reflect diverse physiological characteristics of the organism (which depend on many environmental factors) than a gene implicated in a specific function (and therefore dependent on fewer environmental factors).

### Abiotic influence on Thaumarchaeota (AOA and non-AOA) community distribution

Soil pH and organic matter content were the two main factors associated with global thaumarchaeotal community distribution detected across a wide range of taxonomic resolution, but significant associations were also observed with water content, nitrogen content, C:N ratio, ecosystem type and zinc concentration. Group 1.1c Thaumarchaeota were detected in ~50% of soils, representing all ecosystem types, although more abundant in forest and moorland soils (28% and 71% of Group 1.1c Thaumarchaeota sequences, respectively) than agricultural or grassland ecosystems. This indicates that Group 1.1c are more widely distributed than previously thought, with two clusters predominating. Data confirm their preference for acidic soils (Lehtovirta *et al.*, 2009) but also indicate occurrence of Group 1.1c Thaumarchaeota at lower pH than other acidophilic Thaumarchaeota. In addition, these organisms are mainly present in environments with higher moisture content and organic matter content than AOA, even if they are present in soils over a wide range of water content. A similar pattern was observed for Group 1.3 Thaumarchaeota, which occurred most frequently in forest and moorland soils (53% and 47%, respectively) with lower pH, higher water content and higher organic matter content. The thaumarchaeotal 16S rRNA primers used in this study indicated low relative abundance of this group. Surprisingly, only one metal (Zn) had a significant association with global thaumarchaeotal phylogeny (Table 1) and Group 1.1c and Group 1.3 clusters were present in soils with lower Zn concentration than for AOA, suggesting a possible high susceptibility to Zn level for these organisms.

### Phylogenetic congruence in Thaumarchaeota

A previous study, performed at a low phylogenetic resolution (delineating Group 1.1a, Group 1.1b and Group 1.1a-associated, that is, ~70% identity), showed congruence between 16S rRNA and *amoA* genes from environmental samples (Nicol *et al.*, 2008). The present study demonstrated this congruence at much finer phylogenetic resolution (at least 85% identity based on *amoA*). Moreover, this is the first (to the authors' knowledge) study to test the significance of congruence between 16S rRNA and *amoA* genes of Thaumarchaeota using a statistical tool over large data sets. The findings support the hypothesis of low horizontal gene transfer between Thaumarchaeota (at least with respect to 16S rRNA and *amoA* genes). Comparison of the relative position of these genes on available thaumarchaeotal closed genomes reveals that they are not usually spatially close to each other (Supplementary Figure S5) and the congruence observed is therefore not due to a hitchhiking process.

Congruence between core (housekeeping) and modular (functional) genes also suggests that the ecophysiology of AOA is very similar between strains in the same phylogenetic cluster. This idea of ecological coherence would extend to factors other than pH with specialisation to other ecological niches. Many tools are available for testing congruence using genomic data (Legendre *et al.*, 2002; Hommola *et al.*, 2009; Leigh *et al.*, 2011; Balbuena *et al.*, 2013; Matzke *et al.*, 2014) and can provide insightful information on the coevolution processes of several genes, but their application to Thaumarchaeota is severely limited by the lack of cultivated organisms and environmental genomes. The present study therefore enables analysis of natural communities and facilitates the comparison of many studies using *amoA* or 16S rRNA genes.

### Choice of marker in future ecological and evolutionary studies

The 16S rRNA gene has become the ‘gold standard' for taxonomy and analysis of microbial community structure (Case *et al.*, 2007), but functional or housekeeping genes can be more appropriate for specific applications, because of their potential for use in evolutionary studies and single copy number, in contrast to 16S rRNA genes, which often present intragenomic heterogeneity causing an overestimation of prokaryotic diversity (Sun *et al.*, 2013). Multiple copies of 16S rRNA genes in bacteria and archaea generally have a high level of genetic similarity (Coenye and Vandamme, 2003; Acinas *et al.*, 2004) with differential expression of divergent 16S gene copies possibly depending on environmental conditions (Lopez-Lopez *et al.*, 2007; Gunderson *et al.*, 1987). However, all sequenced members of Thaumarchaeota, Crenarchaeota, Nanoarchaeota or Korarchaeota genomes possess a single 16S rRNA gene (Stoddard *et al.*, 2015), with multiple copies (1–4) reported only in Euryarchaeota genomes (Stoddard *et al.*, 2015). Therefore, the existence of a single copy of 16S rRNA gene in all sequenced archaeal TACK group (archaeal phylogenetic group comprising Thaumarchaeota, Aigarchaeota, Crenarchaeota and Korarchaeota phyla) genomes suggests single copies of both 16S rRNA and *amoA* genes (for example, Spang *et al.*, 2012) in all Thaumarchaeota sequenced in the present study.

This study investigated ecological coherence and phylogenetic congruence in Thaumarchaeota and provides the basis for future analyses to study not only the life strategies and evolutionary history of these organisms, but also the ecosystem function of thaumarchaeotal groups that do not appear to perform ammonia oxidation (Weber *et al.*, 2015).

## Figures and Tables

**Table 1:** Significance of environmental factors at different taxonomic scales for thaumarchaeotal (AOA and non-AOA) 16 S rRNA genes, AOA 16 S rRNA genes and AOA *amoA* genes

	*16S*							*16S AOA*							*amoA*						
% identity	70	80	85	90	95	97	100	70	80	85	90	95	97	100	70	80	85	90	95	97	100
pH	0.79	**0.01**	**0.01**	**0.01**	**0.01**	**0.01**	**0.01**	0.43	0.59	**0.01**	**0.01**	**0.01**	**0.01**	**0.01**	0.05	**0.02**	**0.01**	**0.01**	**0.01**	**0.01**	**0.01**
% organic matter	0.54	**0.01**	**0.01**	**0.01**	**0.02**	**0.03**	**0.01**	0.06	**0.03**	**0.03**	**0.03**	0.12	0.20	0.58	0.55	0.33	0.32	0.14	**0.02**	**0.01**	0.06
% carbon	0.61	0.16	0.22	0.12	0.20	0.33	0.31	0.16	0.23	0.30	0.16	0.36	0.56	0.67	0.08	0.38	0.30	0.56	0.11	0.07	0.08
Bulk density	0.10	0.11	0.09	0.08	0.20	0.50	0.19	0.39	0.18	**0.02**	**0.02**	0.11	0.40	0.23	0.33	0.24	0.31	0.32	0.28	0.12	0.11
Water content	0.07	**0.02**	**0.01**	**0.01**	0.13	0.36	0.34	0.13	0.14	**0.04**	**0.02**	0.08	0.34	0.27	0.35	0.41	0.43	0.65	0.08	0.09	0.06
Vegetation	0.06	**0.01**	**0.01**	**0.01**	**0.04**	0.10	0.20	0.16	0.07	0.07	**0.01**	0.11	0.23	0.29	0.29	0.50	0.48	0.58	0.36	0.24	0.35
% nitrogen	0.60	**0.01**	**0.01**	**0.01**	**0.02**	0.15	0.26	0.10	0.09	**0.02**	**0.01**	**0.03**	0.20	0.45	0.08	0.29	0.13	0.06	**0.01**	**0.01**	**0.01**
Nitrogen stock	0.33	**0.02**	**0.02**	**0.03**	0.27	0.54	0.27	0.12	0.19	0.18	0.08	0.25	0.62	0.32	0.09	0.23	0.34	0.50	0.48	0.48	0.26
Mineralisable N stock	0.70	0.55	0.30	0.40	0.24	0.24	0.13	0.70	0.67	0.68	0.61	0.47	0.30	0.26	0.49	0.38	0.24	0.42	0.44	0.31	0.14
C:N	0.48	**0.01**	**0.01**	**0.02**	0.21	0.30	0.39	0.30	0.35	0.22	0.19	0.14	0.58	0.30	0.70	0.64	0.64	0.31	0.19	0.11	0.14
Phosphorus	0.42	0.49	0.56	0.53	0.71	0.59	0.42	0.48	0.62	0.35	0.31	0.62	0.57	0.52	0.64	0.31	0.17	0.12	0.10	0.05	0.08
Total mineralisable N	0.36	0.13	0.29	0.25	0.29	0.64	0.41	0.52	0.21	**0.01**	**0.01**	**0.03**	0.28	0.45	0.10	0.29	0.33	0.19	0.09	**0.03**	0.14
% nitrate	0.30	0.44	**0.04**	**0.04**	0.09	0.18	0.43	0.79	0.51	0.15	0.05	0.20	0.50	0.45	0.18	0.11	0.12	0.18	0.10	0.10	0.13
Air temperature	0.52	0.22	0.28	0.41	0.43	0.54	0.28	0.69	0.69	0.73	0.73	0.59	0.73	0.57	0.58	0.31	0.25	0.43	0.42	0.27	0.08
Rain	0.38	0.42	0.46	0.21	0.29	0.71	0.15	0.67	0.39	0.57	0.48	0.10	0.38	0.30	0.36	0.81	0.67	0.32	0.11	0.06	0.09
Sun	0.25	0.26	0.19	0.30	0.42	0.34	0.51	0.48	0.48	0.53	0.49	0.47	0.53	0.72	0.07	0.30	0.28	0.54	0.48	0.40	0.35
Cd	0.74	0.66	0.77	0.73	0.69	0.20	0.24	0.78	0.82	0.70	0.50	0.56	0.32	0.33	0.21	0.83	0.56	0.78	0.78	0.77	0.21
Cr	0.70	0.68	0.34	0.27	0.60	0.59	0.55	0.63	0.68	0.08	0.08	0.26	0.37	0.42	0.37	0.25	0.31	0.41	0.22	0.18	0.27
Cu	0.73	0.58	0.61	0.68	0.79	0.56	0.45	0.54	0.56	0.59	0.58	0.79	0.49	0.61	0.20	0.68	0.65	0.81	0.66	0.77	0.37
Ni	0.37	0.47	0.45	0.56	0.78	0.61	0.73	0.68	0.51	0.72	0.54	0.64	0.55	0.65	0.36	0.59	0.59	0.46	0.63	0.45	0.21
Pb	0.68	0.70	0.69	0.72	0.73	0.72	0.63	0.71	0.78	0.45	0.70	0.71	0.79	0.76	0.44	0.66	0.79	0.85	0.91	0.85	0.62
Zn	0.06	**0.03**	**0.01**	**0.01**	**0.01**	0.06	0.22	0.35	0.08	**0.04**	**0.01**	**0.01**	**0.04**	0.28	0.23	0.54	0.41	0.48	0.15	**0.03**	0.21
Al	0.70	0.71	0.15	0.17	0.45	0.76	0.69	0.61	0.67	0.06	0.16	0.50	0.76	0.80	0.30	0.33	0.36	0.55	0.52	0.47	**0.03**
Ti	0.10	0.07	0.23	0.36	0.40	0.51	0.59	0.27	0.08	0.40	0.07	0.22	0.50	0.65	0.09	0.32	0.35	0.41	0.23	0.25	0.14
Mn	0.82	0.46	0.59	0.60	0.72	0.25	0.16	0.64	0.63	0.77	0.36	0.62	0.37	0.28	0.07	0.48	0.28	0.38	0.28	0.30	0.14
As	0.69	0.48	0.13	0.16	0.46	0.49	0.10	0.52	0.57	**0.03**	**0.02**	0.46	0.74	0.23	0.28	0.41	0.49	0.68	0.85	0.74	0.60
Se	0.37	0.52	0.56	0.57	0.69	0.86	0.93	0.75	0.71	0.49	0.49	0.75	0.85	0.91	0.83	0.80	0.82	0.95	0.88	0.74	0.31
Mo	0.37	0.16	0.25	0.27	0.69	0.68	0.68	0.33	0.37	0.21	0.12	0.08	0.17	0.64	0.31	0.59	0.50	0.48	0.27	0.13	0.22
Hg	0.76	0.66	0.26	0.30	0.46	0.26	0.22	0.64	0.68	0.45	0.24	0.46	0.38	0.41	0.13	0.43	0.51	0.71	0.83	0.76	0.55
No. of clusters	4	13	18	24	47	76	348	4	11	15	19	32	47	162	3	11	18	28	59	84	290

The number of clusters is indicated for each taxonomic resolution. Numbers in bold indicate where the environmental variable of interest explains the microbial community structure at a specific taxonomic scale. The different parameters measured are: pH, % organic matter (loss on ignition), % carbon (C stock loss on ignition), bulk density (g cm^−3^), soil moisture content (% H_2_O), % nitrogen (proportion of the nitrogen content (mg N kg^−1^ dry soil)), nitrogen stock (measured on the top surface zone (0–15 m) (t ha^−1^)), C:N ratio, phosphorous (Olsen PO_4_ mg kg^−1^), total mineralisable nitrogen (total mineral (NO_3_^−^+NH_4_^+^) nitrogen concentration (mg N kg^−1^ dry soil)), mineralisable nitrogen stock (total mineral (NO_3_^−^+NH_4_^+^) nitrogen stock (kg N ha^−1^)), nitrate proportion of the mineralisable nitrogen stock (% nitrate), vegetation (agricultural, grassland, forest or moorland), mean air annual temperature (°C), mean monthly rainfall (mm month^−1^), average hours of sun per day and the concentration (mg kg^−1^) of 12 metals (Cd, Cr, Cu, Ni, Pb, Zn, Al, Ti, Mn, As, Se and Mo).
