# Supplementary material for: Phylogenetic congruence and ecological coherence in terrestrial Thaumarchaeota
Source: ISME J. 2015 Jul 3;10(1):85–96. doi: 10.1038/ismej.2015.101 (PMC4604658; doi:10.1038/ismej.2015.101)
Supplement: Supplementary Information [file ismej2015101x1.doc]

# **Supporting Information:** **Phylogenetic congruence and ecological coherence in terrestrial Thaumarchaeota**

# SI Material and Methods

# *High-throughput sequencing*

# The 454 high-throughput sequencing was performed on each soil for both *amoA* and 16S rRNA genes. Triplicate PCRs were performed with primers specific for Thaumarchaeota, extended as amplicon fusion primers with respective primer A and B adapters, key sequences and one of 24 multiplex identifiers as recommended by Roche, with 24 different barcode decamers enabling bidirectional amplicon sequencing. Amplifications were performed in triplicate, before pooling, using primers CrenamoA23f and CrenamoA616r (Tourna *et al.*, 2008) for *amoA* and A109f (Grosskopf et al., 1998) and 752r (reverse complement of 771f (Ochsenreiter *et al*., 2003)) for 16S rRNA. Amplification of *amoA* was unsuccessful in four CS soils despite several attempts, potentially due to low abundance or absence of ammonia oxidizing archaea. Amplicons were cleaned using NucleoSpin Extract II Kit (Fisher) and further purified with AMpure beads (Beckman Coulter). Amplicons were quantified using PicoGreen (Life Technologies) to pool samples in equimolar ratio for sequencing. High-throughput sequencing was performed on a second-generation pyrosequencer (454 GS FLX Titanium; Roche) using titanium reagents and titanium procedures as recommended by the manufacturer.

# Pyrosequencing reads were quality filtered using the automatic amplicon pipeline of the GS Run Processor (Roche) and cleaned using AmpliconNoise (Quince *et al.*, 2011). This pipeline is designed to remove 454 homopolymer errors, PCR single nucleotide errors and PCR chimeras. After removal of barcodes and dereplication of the reads to unique sequences (with abundance calculated), the unique reverse reads were reversed and complemented. The forward and reverse reads were assembled based on an exact match over at least 100 bp with a custom C program using exact pairwise Needleman–Wunsch alignments (see Gubry-Rangin *et al.*, 2011). Total abundance of each read was the sum of forward and reverse sequences. Unique sequence databases were obtained by dereplication at 100% using Uclust (Edgar 2010) implemented in QIIME (Caporaso *et al*., 2010). Alignment of sequences was checked manually and misaligned regions of the 16S rRNA gene sequence dataset were removed for subsequent phylogenetic analyses. A maximum likelihood (ML) tree was built for the 16S rRNA gene dataset under a GTR with 4-category gamma distribution substitution model using MEGA6 software (Tamura *et al.*, 2013). Sequences forming a deeply separated phylogenetic cluster (tree not shown) were affiliated to the Euryarchaeota phylum following a BLASTn in NCBI and those sequences were removed from subsequent analyses.

# *Bayesian phylogenetic analyses*

# Both *amoA* and 16S rRNA gene datasets were scanned for recombinants using Recombinant Detection Program 4 (RDP4) software (Martin *et al.,* 2010) with a combination of the RDP, GENECONV, MaxChi and BootScan methods. After manual curation of these results, sequences detected as recombinant were removed from subsequent phylogenetic steps. Phylogenetic analyses were performed using a Bayesian Markov Chain Monte Carlo approach (MCMC) using BEAST software package version 1.8.0 (Drummond *et al.* 2012). Codon saturation was tested (Xia *et al.*, 2013) and only detected for *amoA*, resulting in the removal of the third codon position for this dataset. PartitionFinder (Lanfear *et al.* 2012) determined SYM as the best substitution model for both genes among the BEAST/Beauti models available. Site Heterogeneity Model was set to ‘Gamma’ for the archaeal *amoA* gene and to ‘Gamma + Invariant Sites’ for the 16S rRNA gene. Both analyses were performed using 4 gamma categories and without codon partitioning. An uncorrelated lognormal relaxed clock model was used with a Yule process for both genes. A UPGMA starting tree was created with a 200 and 270 million chain lengths for 16S rRNA and *amoA* genes, respectively. Analyses were performed two or three times per dataset and results were combined using the LogCombiner tool when the log likelihood of the run had converged and Effective Sample Size was >200. Combined trees were converted into a single tree using TreeAnnotator with a 10% burnin of the total number of trees, no posterior limit and a maximum clade credibility tree as the target tree keeping the target height of the nodes. Trees were ladderized using package ape (Paradis *et al.* 2004) in R.

**SI figures**

**
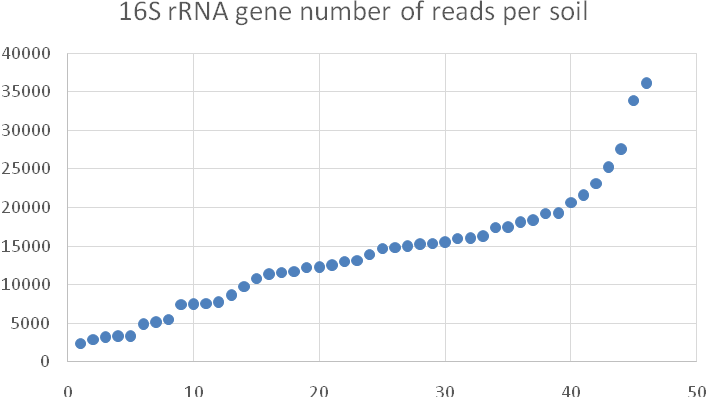

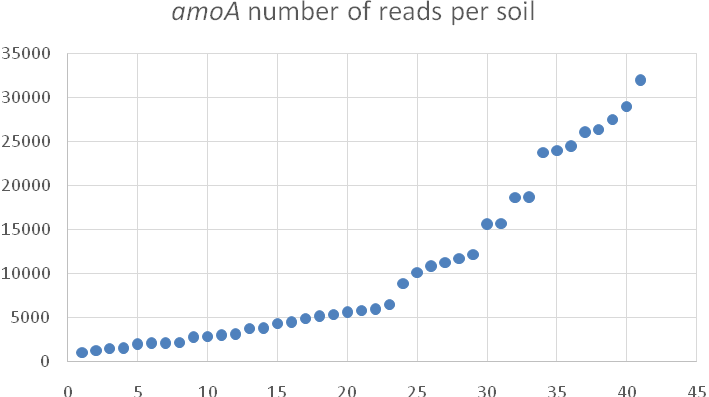
**

**Figure. S1.** Representation of the number of reads (after noise removal) per soil for 16S rRNA and *amoA* gene datasets.

**

Figure. S2.** Cladogram representation of the 16S rRNA gene phylogenetic tree. The number of reads (in brackets), pH preference (circle), water content preference (diamond), organic matter preference (triangle) and number of reads (black bars) are indicated next to each defined clusters (in bold). A gradient of colours reflects levels of environmental preferences, with arbitrary limits between the different classes.

**

**

**Figure. S3.** Cladogram representation of the *amoA* phylogenetic tree. Legend is as described in Figure S2, except that one cluster does not contain environmental preferences as it contained only one culture sequence.

**
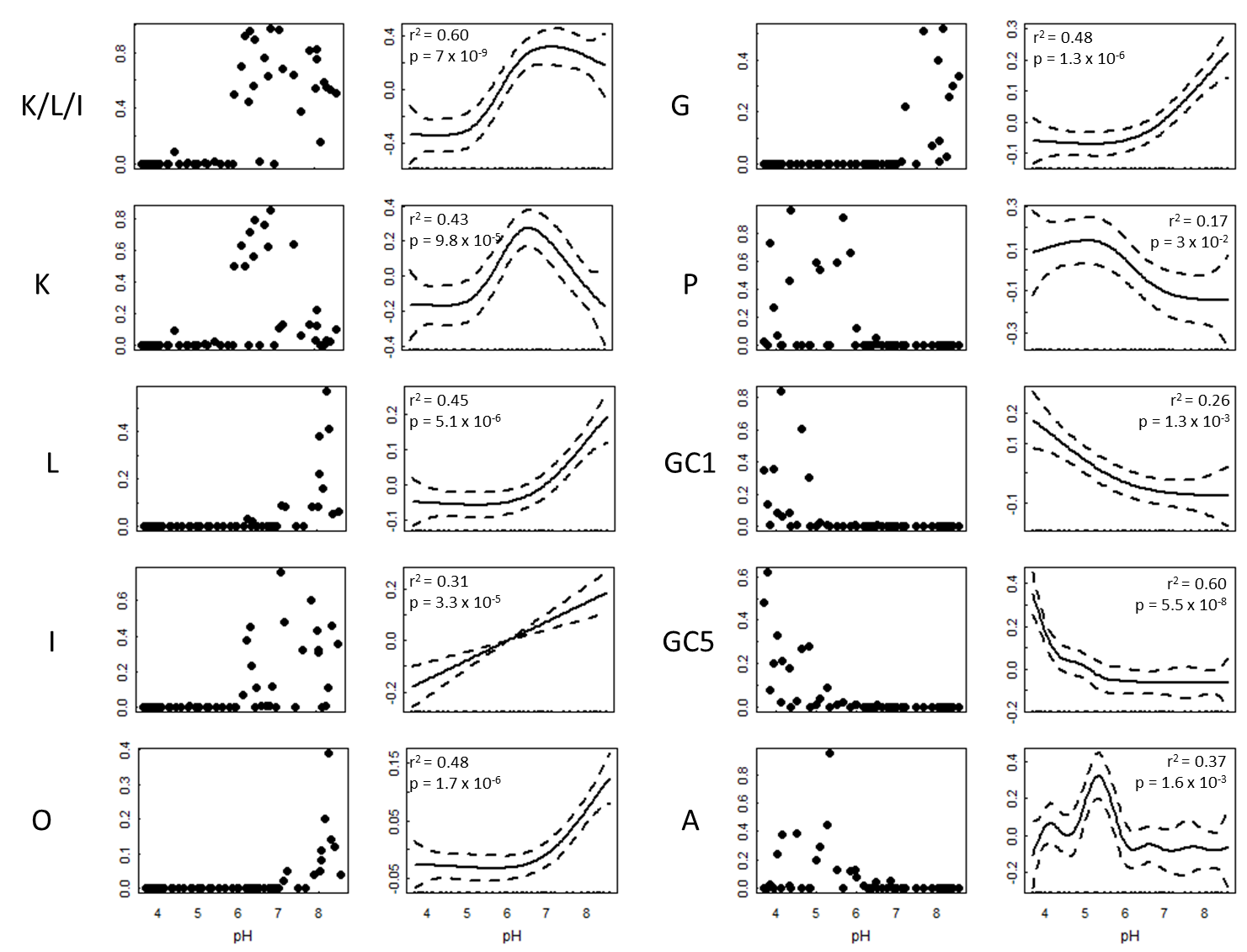
**

**Figure. S4.** The relative abundance of sequences within each of the nine most abundant clusters of 16S rRNA gene clusters is represented as a function of soil pH. The first column represents the percentage relative abundance and the second column the best-fitting model of this distribution according to a generalised additive modelling. The regression coefficients and associated *p*-values are provided. Representation of the grouped clusters K, L and I is based on the congruence analysis.

**
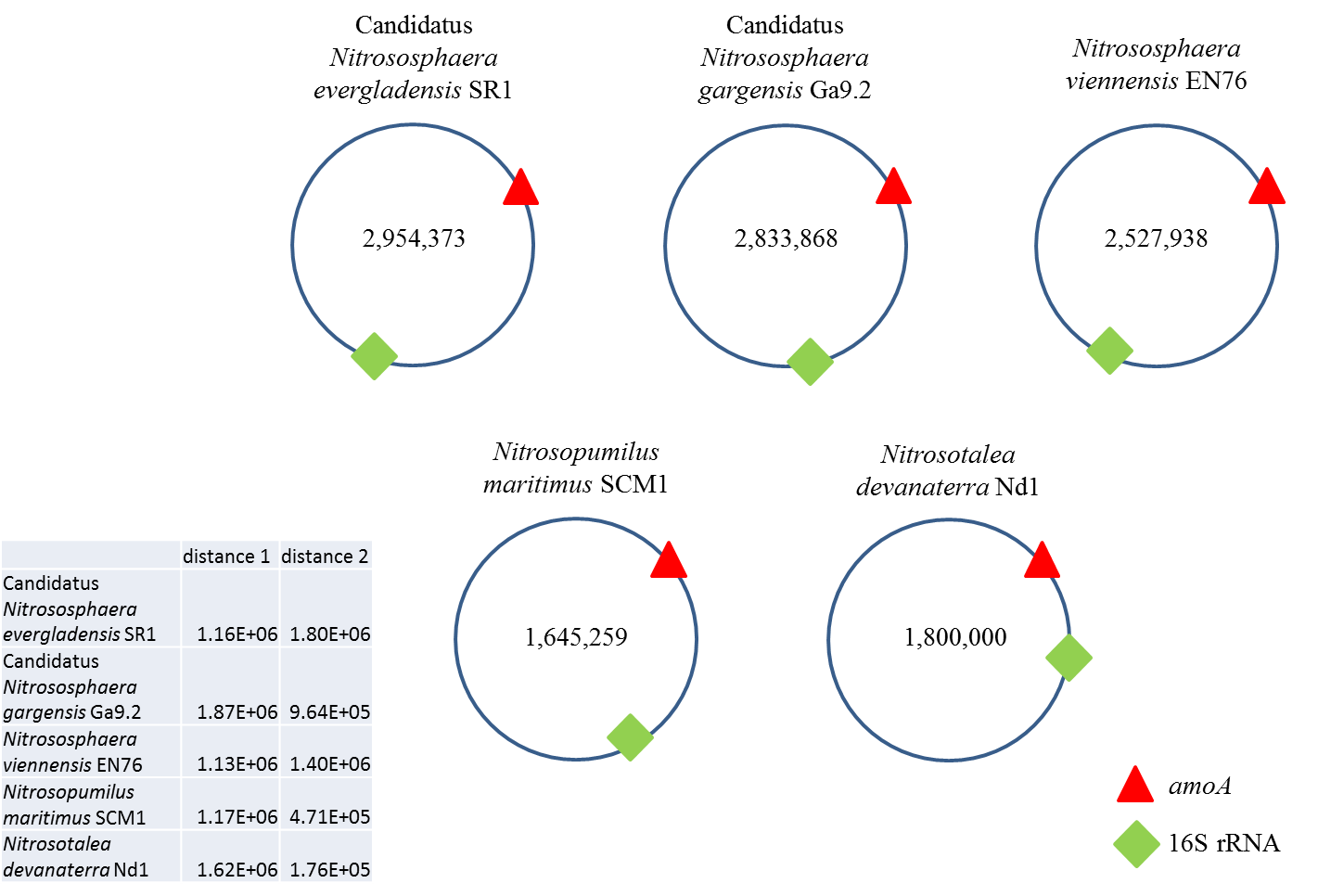
**

**Figure S5.** Schematic representation of the relative position of *amoA* and 16S rRNA genes on five available AOA closed full genomes. Total genome size and respective distance between the two genes (bp) are indicated for each organism.

**SI tables**

Table S1: Contextual data for the soils used in this study. The different parameters measured are: pH, % organic matter (loss on ignition (LOI)), % carbon (C stock LOI), bulk density, soil moisture content (% H_2_O), % nitrogen (proportion of the nitrogen content (mg N kg^-1^ dry soil)), nitrogen stock (measured on the top surface zone (0-15 m) (t/ha)), C:N ratio, phosphorous (Olsen PO_4_ mg kg^−1^), total mineralisable nitrogen (total mineral (NO_3_^-^+NH_4_^+^) nitrogen concentration (mg N kg^-1^ dry soil)), mineralisable nitrogen stock (total mineral (NO_3_^-^+NH_4_^+^) nitrogen stock (kg N ha^-1^)), nitrate proportion of the mineralisable nitrogen stock (% nitrate), vegetation (agricultural, grassland, forest or moorland), mean air annual temperature (^o^C), mean monthly rainfall (mm month^-1^), average hours of sun per day and the concentration (mg kg^−1^) of twelve metals (Cd, Cr, Cu, Ni, Pb, Zn, Al, Ti, Mn, As, Se and Mo). “nd” indicates that data were not determined.

| **Soil ID** | **pH** | **% organic matter** | **% carbon** | **bulk density** | **Water content** | **% nitrogen** | **nitrogen stock** | **C:N** | **phosphorus** | **Total mineralisable N** | **mineralisable N stock** | **% nitrate** | **vegetation** | **air temperature** | **rain** | **sun** | **Cd** | **Cr** | **Cu** | **Ni** | **Pb** | **Zn** | **Al** | **Ti** | **Mn** | **As** | **Se** | **Mo** | **Hg** |
| --- | --- | --- | --- | --- | --- | --- | --- | --- | --- | --- | --- | --- | --- | --- | --- | --- | --- | --- | --- | --- | --- | --- | --- | --- | --- | --- | --- | --- | --- |
| **357** | 3.7 | 15 | 60 | 0.5 | 40 | 0.4 | 2.6 | 22 | 9 | 21 | 15 | 0.9 | F | 9 | 56 | 4 | 0.1 | 2 | 7 | 2 | 58 | 21 | 883 | 15 | 18 | 2 | 0.9 | 0.5 | 0.0 |
| **540** | 3.8 | 10 | 79 | 0.9 | 33 | 0.2 | 3.1 | 31 | 39 | 3 | 4 | 0.8 | F | 8 | 60 | 3 | 0.1 | 7 | 6 | 3 | 57 | 9 | 2710 | 10 | 8 | 5 | 1.1 | 0.5 | 0.1 |
| **678** | 3.9 | 59 | 99 | 0.2 | 80 | 1.6 | 4.8 | 20 | 33 | 12 | 4 | 0.1 | M | 5 | 119 | 3 | 0.6 | 14 | 15 | 7 | 159 | 52 | 5320 | 33 | 92 | 10 | 2.6 | 0.8 | 0.2 |
| **442** | 3.9 | 20 | 115 | 0.7 | 40 | 0.5 | 4.7 | 26 | 7 | 18 | 18 | 0.0 | M | 9 | 94 | 4 | 0.2 | 6 | 24 | 3 | 116 | 13 | 1990 | 21 | 11 | 15 | 1.7 | 2.9 | 0.1 |
| **139** | 4.0 | 13 | 73 | 0.7 | 33 | 0.4 | 3.7 | 18 | 4 | 18 | 18 | 1.0 | F | 9 | 110 | 4 | 0.1 | 8 | 19 | 7 | 53 | 51 | 3310 | 11 | 170 | 30 | 1.9 | 0.8 | 0.1 |
| **1075** | 4.1 | 89 | 137 | 0.2 | 80 | 1.6 | 4.4 | 32 | 42 | 6 | 2 | 0.0 | M | 8 | 83 | 3 | 0.3 | 2 | 2 | 2 | 27 | 17 | 2180 | 57 | 17 | 2 | 2.8 | 0.7 | 0.2 |
| **136** | 4.2 | 6 | 33 | 0.7 | 33 | 0.2 | 1.8 | 19 | 3 | 4 | 5 | 1.0 | F | 9 | 110 | 4 | 0.2 | 12 | 9 | 18 | 21 | 84 | 8110 | 15 | 462 | 11 | 1.4 | 0.5 | 0.0 |
| **334** | 4.3 | 85 | 63 | 0.1 | 92 | 2.6 | 3.4 | 19 | 14 | 24 | 3 | 0.1 | M | 8 | 167 | 3 | 0.3 | 5 | 16 | 5 | 78 | 31 | 4530 | 104 | 32 | 21 | 4.4 | 1.2 | 0.3 |
| **13** | 4.4 | 7 | 56 | 1.0 | 27 | 0.4 | 6.0 | 8 | 34 | 19 | 29 | 0.9 | G | 10 | 103 | 4 | 0.1 | 14 | 16 | 14 | 24 | 53 | 9390 | 21 | 657 | 14 | 1.4 | 0.6 | 0.0 |
| **724** | 4.6 | 62 | 114 | 0.2 | 78 | 1.2 | 3.9 | 28 | 22 | 14 | 5 | 0.0 | M | 9 | 108 | 4 | 0.2 | 7 | 11 | 4 | 56 | 27 | 3380 | 249 | 60 | 3 | 2.0 | 1.1 | 0.1 |
| **899** | 4.8 | 95 | 79 | 0.1 | 92 | 1.5 | 2.2 | 34 | 29 | 19 | 3 | 0.0 | M | 9 | 125 | 4 | 0.3 | 1 | 7 | 2 | 37 | 29 | 468 | 30 | 13 | 3 | 3.6 | 0.8 | 0.2 |
| **26** | 4.8 | 5 | 53 | 1.3 | 23 | 0.2 | 4.3 | 10 | 7 | 33 | 65 | 0.8 | G | 11 | 76 | 4 | 0.1 | 16 | 15 | 14 | 32 | 45 | 8730 | 45 | 878 | 11 | 1.0 | 0.9 | 0.0 |
| **233** | 5.1 | 8 | 65 | 1.0 | 16 | 0.4 | 5.2 | 12 | 17 | 16 | 23 | 1.0 | F | 10 | 53 | 4 | 0.2 | 19 | 15 | 18 | 82 | 92 | 12200 | 92 | 897 | 7 | 1.2 | 0.8 | 0.2 |
| **806** | 5.3 | 6 | 51 | 1.1 | 26 | 0.3 | 4.6 | 12 | 105 | 12 | 19 | 0.9 | F | 9 | 58 | 4 | 0.1 | 19 | 27 | 13 | 62 | 97 | 16200 | 112 | 1010 | 8 | 0.8 | 0.5 | 0.1 |
| **279** | 5.3 | 5 | 51 | 1.2 | 14 | 0.2 | 3.7 | 11 | 38 | 24 | 44 | 1.0 | G | 9 | 54 | 4 | 0.2 | 12 | 19 | 10 | 38 | 81 | 6760 | 41 | 465 | 5 | 0.4 | 1.6 | 0.0 |
| **207** | 5.7 | 24 | 75 | 0.4 | 70 | 1.1 | 6.3 | 12 | 25 | 14 | 8 | 1.0 | F | 9 | 105 | 4 | 0.3 | 21 | 31 | 21 | 43 | 62 | 15100 | 15 | 173 | 10 | 2.7 | 1.9 | 0.1 |
| **534** | 5.8 | 10 | 79 | 0.9 | 30 | 0.4 | 6.0 | 12 | 22 | 12 | 16 | 0.7 | G | 7 | 152 | 3 | 0.1 | 26 | 9 | 8 | 40 | 47 | 8760 | 20 | 606 | 7 | 1.7 | 0.7 | 0.1 |
| **887** | 6.0 | 12 | 57 | 0.6 | 53 | 0.5 | 4.0 | 15 | 3 | 10 | 9 | 0.5 | M | 7 | 71 | 4 | 0.4 | 20 | 12 | 26 | 57 | 62 | 10600 | 332 | 654 | 3 | 2.2 | 4.8 | 0.1 |
| **448** | 6.2 | 6 | 50 | 1.0 | 29 | 0.3 | 4.9 | 10 | 11 | 5 | 8 | 0.4 | G | 9 | 68 | 4 | 0.2 | 23 | 31 | 20 | 34 | 103 | 13400 | 6 | 782 | 10 | 0.7 | 1.3 | 0.0 |
| **715** | 6.3 | 3 | 33 | 1.2 | 18 | 0.2 | 2.9 | 9 | 9 | 11 | 21 | 0.9 | A | 8 | 54 | 4 | 0.1 | 30 | 9 | 27 | 16 | 68 | 10800 | 121 | 864 | 3 | 0.5 | 0.4 | 0.0 |
| **761** | 6.4 | 5 | 59 | 1.4 | 14 | 0.2 | 4.3 | 16 | 35 | 9 | 18 | 1.0 | A | 8 | 63 | 4 | 0.4 | 21 | 36 | 22 | 99 | 146 | 9810 | 76 | 624 | 8 | 1.0 | 0.9 | 0.5 |
| **343** | 6.4 | 10 | 64 | 0.8 | 35 | 0.5 | 6.2 | 10 | 46 | 7 | 8 | 0.8 | G | 8 | 97 | 3 | 0.3 | 36 | 21 | 24 | 45 | 142 | 24200 | 16 | 1120 | 18 | 1.6 | 0.7 | 0.0 |
| **308** | 6.5 | 6 | 59 | 1.1 | 18 | 0.3 | 5.7 | 11 | 6 | 19 | 32 | 0.9 | F | 9 | 59 | 4 | 0.3 | 17 | 19 | 19 | 35 | 78 | 9320 | 28 | 587 | 9 | 0.8 | 0.6 | 0.0 |
| **684** | 6.6 | 13 | 50 | 0.5 | 41 | 0.6 | 3.8 | 14 | 65 | 18 | 12 | 1.0 | A | 8 | 56 | 3 | 0.2 | 20 | 16 | 8 | 30 | 38 | 13400 | 28 | 243 | 6 | 1.2 | 0.3 | 0.1 |
| **707** | 6.8 | 5 | 46 | 1.2 | 30 | 0.3 | 4.4 | 10 | 39 | 4 | 7 | 0.2 | G | 7 | 77 | 3 | 0.2 | 32 | 17 | 32 | 24 | 80 | 13600 | 87 | 610 | 15 | 0.7 | 0.3 | 0.0 |
| **910** | 6.9 | 3 | 36 | 1.3 | 16 | 0.2 | 3.2 | 11 | 68 | 3 | 6 | 0.3 | G | 8 | 61 | 4 | 0.1 | 10 | 4 | 7 | 10 | 16 | 3560 | 112 | 86 | 6 | 0.4 | 0.2 | 0.0 |
| **566** | 6.9 | 4 | 48 | 1.3 | 17 | 0.2 | 3.9 | 12 | 8 | 11 | 22 | 1.0 | A | 8 | 54 | 4 | 0.2 | 18 | 19 | 11 | 50 | 75 | 10300 | 29 | 402 | 7 | 1.1 | 0.4 | 0.1 |
| **168** | 7.1 | 14 | 86 | 0.8 | 40 | 0.5 | 6.2 | 12 | 5 | 18 | 21 | 0.9 | F | 9 | 60 | 4 | 0.6 | 38 | 16 | 33 | 76 | 133 | 27000 | 26 | 2070 | 19 | 2.9 | 2.8 | 0.1 |
| **759** | 7.2 | 5 | 49 | 1.3 | 16 | 0.2 | 3.3 | 15 | 68 | 13 | 25 | 1.0 | G | 8 | 63 | 4 | 0.4 | 22 | 34 | 27 | 86 | 170 | 8140 | 70 | 612 | 12 | 0.9 | 0.6 | 0.2 |
| **149** | 7.7 | 11 | 89 | 1.0 | 34 | 0.6 | 8.1 | 9 | 26 | 39 | 57 | 0.9 | G | 10 | 68 | 4 | 1.3 | 36 | 29 | 33 | 563 | 745 | 19100 | 25 | 1460 | 58 | 1.8 | 2.1 | 0.1 |
| **205** | 7.9 | 4 | 47 | 1.4 | 18 | 0.2 | 3.8 | 11 | 16 | 6 | 13 | 1.0 | F | 10 | 47 | 4 | 0.8 | 20 | 31 | 22 | 88 | 190 | 8110 | 26 | 363 | 11 | 1.6 | 1.3 | 0.0 |
| **511** | 8.0 | 5 | 53 | 1.3 | 21 | 0.2 | 4.2 | 11 | 4 | 1 | 3 | 0.6 | A | 9 | 65 | 4 | 1.1 | 25 | 91 | 18 | 57 | 174 | 11100 | 31 | 2910 | 7 | 1.2 | 0.9 | 0.0 |
| **490** | 8.1 | 5 | 76 | 1.8 | 21 | 0.2 | 6.3 | 11 | 40 | 9 | 26 | 0.7 | A | 9 | 48 | 4 | 0.2 | 28 | 28 | 22 | 43 | 105 | 12500 | 59 | 1360 | 9 | 0.9 | 1.3 | 0.0 |
| **159** | 8.1 | 6 | 52 | 1.1 | 23 | 0.3 | 5.0 | 9 | 21 | 19 | 31 | 0.9 | G | 10 | 44 | 4 | 0.1 | 24 | 17 | 16 | 28 | 65 | 12000 | 23 | 205 | 8 | 0.9 | 0.4 | 0.0 |
| **433** | 8.2 | 4 | 43 | 1.4 | 20 | 0.2 | 3.8 | 9 | 20 | 2 | 5 | 0.4 | G | 9 | 49 | 4 | 0.2 | 21 | 11 | 16 | 21 | 53 | 10000 | 10 | 343 | 9 | 0.6 | 0.6 | 0.0 |
| **78** | 8.3 | 24 | 117 | 0.6 | 48 | 1.3 | 11.3 | 15 | 18 | 16 | 14 | 0.1 | G | 10 | 79 | 4 | 0.6 | 7 | 8 | 6 | 38 | 80 | 4720 | 19 | 723 | 9 | 2.8 | 0.3 | 0.1 |
| **69** | 8.3 | 23 | 81 | 0.4 | 46 | 1.2 | 7.4 | 15 | 16 | 16 | 10 | 0.4 | F | 9 | 75 | 4 | 0.4 | 8 | 8 | 14 | 38 | 84 | 4300 | 10 | 781 | 9 | 0.9 | 0.4 | 0.1 |
| **251** | 8.4 | 6 | 63 | 1.2 | 25 | 0.3 | 5.9 | 12 | 10 | 10 | 18 | 0.9 | G | 10 | 50 | 4 | 0.2 | 17 | 9 | 12 | 20 | 56 | 8520 | 39 | 504 | 10 | 1.0 | 0.6 | 0.0 |
| **531** | 8.6 | 6 | 64 | 1.3 | 27 | 0.4 | 6.6 | 15 | 40 | 14 | 26 | 1.0 | A | 8 | 59 | 4 | 0.4 | 12 | 12 | 16 | 31 | 64 | 6520 | 22 | 945 | 9 | 0.4 | 0.4 | 0.0 |
| **Cs4.5** | 4.5 | 12 | nd | nd | 30 | nd | 0.4 | 19 | nd | 15 | nd | 1.0 | A | nd | nd | nd | nd | nd | nd | nd | nd | nd | nd | nd | nd | nd | nd | nd | nd |
| **Cs5** | 5.0 | 11 | nd | nd | 31 | nd | 0.4 | 18 | nd | 14 | nd | 1.0 | A | nd | nd | nd | nd | nd | nd | nd | nd | nd | nd | nd | nd | nd | nd | nd | nd |
| **Cs5.5** | 5.5 | 13 | nd | nd | 31 | nd | 0.4 | 18 | nd | 10 | nd | 1.0 | A | nd | nd | nd | nd | nd | nd | nd | nd | nd | nd | nd | nd | nd | nd | nd | nd |
| **Cs6** | 6.0 | 11 | nd | nd | 30 | nd | 0.3 | 21 | nd | 6 | nd | 0.9 | A | nd | nd | nd | nd | nd | nd | nd | nd | nd | nd | nd | nd | nd | nd | nd | nd |
| **Cs6.5** | 6.5 | 11 | nd | nd | 29 | nd | 0.3 | 22 | nd | 5 | nd | 0.9 | A | nd | nd | nd | nd | nd | nd | nd | nd | nd | nd | nd | nd | nd | nd | nd | nd |
| **Cs7** | 7.0 | 14 | nd | nd | 31 | nd | 0.4 | 22 | nd | 5 | nd | 0.9 | A | nd | nd | nd | nd | nd | nd | nd | nd | nd | nd | nd | nd | nd | nd | nd | nd |
| **Cs7.5** | 7.5 | 12 | nd | nd | 29 | nd | 0.3 | 21 | nd | 5 | nd | 0.8 | A | nd | nd | nd | nd | nd | nd | nd | nd | nd | nd | nd | nd | nd | nd | nd | nd |

**Table S2:** Available ammonia oxidising cultures or genomic fragments presenting both *amoA* and 16S rRNA gene sequences. These sequences were used in the phylogenetic analysis.

| Organism | Strain | Origin | Reference |
| --- | --- | --- | --- |
| *Nitrososphaera gargensis* | Ga9.2 | Hot spring | Spang *et al.*, 2012 |
| *Nitrosopumilus* sp. | SJ | Marine sediment | Park *et al.*, 2010 |
| *Nitrososphaera viennensis* | EN76 | Garden soil | Tourna *et al.* , 2011 |
| *Nitrosotalea devanaterra* | Nd1 | Agricultural soil | Lehtovirta-Morley *et al.*, 2011 |
| *Nitrosoarchaeum koreensis* | MY1 | Agricultural soil | Kim *et al.*, 2011 |
| *Nitrosopumilus* sp. | NM25 | Coastal sand of an eelgrass zone | Matsutani *et al.*, 2011 |
| *Nitrosopumilus* sp. | 2 strains | Marine water column | Santoro & Casciotti, 2011 |
| *Nitrosoarchaeum limnia* | SFB1 | Estuary sediment | Blainey *et al.*, 2011 |
| *Nitrosoarchaeum limnia* | BG20 | Estuary sediment | Mosier *et al.*, 2012a |
| *Nitrosopumilus salaria* | BD31 | Estuary sediment | Mosier *et al.*, 2012b |
| *Nitrosopumilus koreensis* | AR1 | Marine sediment | Park *et al.*, 2012a |
| *Nitrosopumilus sediminis* | AR2 | Marine sediment | Park *et al.*, 2012b |
| *Nitrosotenuis* sp. | 3 strains | Freshwater | French *et al.*, 2012 |
| *Nitrososphaera* sp. | JG1 | Agricultural soil | Kim *et al.*, 2012 |
| *Nitrosotenuis uzonensis* | N4 | Hot spring | Lebedeva *et al.*, 2013 |
| *Nitrosotenuis* sp. | MY2 | Agricultural soil | Jung *et al.*, 2013 |
| *Nitrososphaera* sp. | MY3 | Contaminated soil | Jung *et al.*, 2013 |
| *Nitrosotalea* sp. | CS | Acidic mine | Jung *et al.*, 2013 |
| Genomic fragment | 54d9 | Soil | Treusch *et al.*, 2005 |

**Table S3.** Spearman's rank-order correlation test for significant variables for the 16S rRNA gene **(a)** and *amoA* datasets **(b)**. Upper part of the matrix corresponds to Spearman's rho and lower part corresponds to the respective *p* value. Correlation values in bold are significantly correlated and significant *p* values are indicated in bold with an asterisk (*p* value <0.05).

|  | **(a)** | | | | | | | | | | | | | | | | | | | | | | | | |  |  | | |  | | |  | | |  | | |
| --- | --- | --- | --- | --- | --- | --- | --- | --- | --- | --- | --- | --- | --- | --- | --- | --- | --- | --- | --- | --- | --- | --- | --- | --- | --- | --- | --- | --- | --- | --- | --- | --- | --- | --- | --- | --- | --- | --- |
| **16S rRNA** | | pH | | LOI | Moisture | | | | %N | | | | N stock | | | | C:N | | Nitrate | | | Zn | |  | | | | | | | | | | | | | | |
| pH | |  | | **-0.411** | **-0.366** | | | | -0.237 | | | | **0.440** | | | | **-0.460** | | 0.118 | | | **0.540** | |  | | | | | | | | | | | | | | |
| LOI | | **0.010*** | |  | **0.882** | | | | **0.941** | | | | 0.228 | | | | **0.653** | | **-0.398** | | | **-0.405** | |  | | | | | | | | | | | | | | |
| Moisture | | **0.022*** | | **<0.001*** |  | | | | **0.854** | | | | 0.119 | | | | **0.570** | | **-0.509** | | | **-0.415** | |  | | | | | | | | | | | | | | |
| %N | | 0.147 | | **<0.001*** | **<0.001*** | | | |  | | | | **0.418** | | | | **0.436** | | **-0.436** | | | -0.293 | |  | | | | | | | | | | | | | | |
| N stock | | **0.005*** | | 0.162 | 0.469 | | | | **0.008*** | | | |  | | | | -0.277 | | -0.054 | | | **0.346** | |  | | | | | | | | | | | | | | |
| C:N | | **0.003*** | | **<0.001*** | **<0.001*** | | | | **0.006*** | | | | 0.088 | | | |  | | -0.268 | | | **-0.449** | |  | | | | | | | | | | | | | | |
| Nitrate | | 0.473 | | **0.012*** | **0.001*** | | | | **0.006*** | | | | 0.743 | | | | 0.099 | |  | | | **0.411** | |  | | | | | | | | | | | | | | |
| Zn | | **<0.001*** | | **0.011*** | **0.009*** | | | | 0.070 | | | | **0.032*** | | | | **0.004*** | | **0.009*** | | |  | |  | | | | | | | | | | | | | | |
|  | | |  | | | |  | | | | |  | | | |  | | | | |  | | | |  | | |  | | |  | | |  | | |  | |
|  |  | | | | | | | | | | | | | | | | | | | | | | | | |  | | |  | | |  | | |  | | |  |
|  | **(b)** | | | | | | | | | | | | | | | | | | | | | | | | |  | | |  | | |  | | |  | | |  |
| ***amoA*** | | pH | | LOI | | %N | | | | N mineralized | | | | | Zn | | | Al | |  | | | | | | | | | | | | | | | | | | |
| pH | |  | | -0.255 | | -0.128 | | | | -0.265 | | | | | 0.363 | | | 0.140 | |  | | | | | | | | | | | | | | | | | | |
| LOI | | 0.145 | |  | | **0.951** | | | | **0.508** | | | | | -0.237 | | | -0.084 | |  | | | | | | | | | | | | | | | | | | |
| %N | | 0.472 | | **<0.001*** | |  | | | | **0.485** | | | | | -0.206 | | | 0.051 | |  | | | | | | | | | | | | | | | | | | |
| N mineralized | | 0.130 | | **0.002*** | | **0.004*** | | | |  | | | | | -0.260 | | | -0.154 | |  | | | | | | | | | | | | | | | | | | |
| Zn | | **0.036*** | | 0.177 | | 0.243 | | | | 0.137 | | | | |  | | | **0.459** | |  | | | | | | | | | | | | | | | | | | |
| Al | | 0.430 | | 0.638 | | 0.774 | | | | 0.384 | | | | | **0.006*** | | |  | |  | | | | | | | | | | | | | | | | | | |
|  | | | | | | | |  | | |  | | |  | | | | | |  | | |  | | |  |  | | |  | | |  | | |  | | |

**Supplementary references:**

Blainey PC, Mosier AC, Potanina A, Francis CA, Quake SR. (2011). Genome of a low-salinity ammonia-oxidizing archaeon determined by single-cell and metagenomic analysis. PLoS One 6(2):e16626.

Caporaso JG, Kuczynski J, Stombaugh J, Bittinger K, Bushman FD, Costello EK *et al*. (2010). QIIME allows analysis of high-throughput community sequencing data. Nature Methods 7(5):335-336.

Drummond AJ, Suchard MA, Xie D, Rambaut A. (2012). Bayesian phylogenetics with BEAUti and the BEAST 1.7. Mol Biol Evol 29(8):1969–1973.

Edgar RC. (2010). Search and clustering orders of magnitude faster than BLAST. Bioinformatics 26(19):2460–2461.

French E, Kozlowski JA, Mukherjee M, Bullerjahn G, Bollmann A. (2012). Ecophysiological characterization of ammonia-oxidising archaea and bacteria from freshwater. Appl Environ Microbiol 78(16):5773–5780.

Grosskopf R, Stubner S & Liesack W. (1998) Novel euryarchaeotal lineages detected on rice roots and in the anoxic bulk soil of flooded rice microcosms. Appl Environ Microbiol 64(12):4983–4989.

[Gubry-Rangin C](http://www.ncbi.nlm.nih.gov/pubmed/?term=Gubry-Rangin C%5BAuthor%5D&cauthor=true&cauthor_uid=22158986), [Hai B](http://www.ncbi.nlm.nih.gov/pubmed/?term=Hai B%5BAuthor%5D&cauthor=true&cauthor_uid=22158986), [Quince C](http://www.ncbi.nlm.nih.gov/pubmed/?term=Quince C%5BAuthor%5D&cauthor=true&cauthor_uid=22158986), [Engel M](http://www.ncbi.nlm.nih.gov/pubmed/?term=Engel M%5BAuthor%5D&cauthor=true&cauthor_uid=22158986), [Thomson BC](http://www.ncbi.nlm.nih.gov/pubmed/?term=Thomson BC%5BAuthor%5D&cauthor=true&cauthor_uid=22158986), [James P](http://www.ncbi.nlm.nih.gov/pubmed/?term=James P%5BAuthor%5D&cauthor=true&cauthor_uid=22158986) *et al.*. (2011). Niche specialization of terrestrial archaeal ammonia oxidizers. Proc Natl Acad Sci U S A 108(52):21206–21211.

Kim BK, Jung MY, Yu DS, Park SJ, Oh TK, Rhee SK *et al*. (2011). Genome sequence of an ammonia-oxidising soil archaeon, “Candidatus *Nitrosoarchaeum koreensis*” MY1. J. Bacteriol 193(19):5539–5540.

Kim JG, [Jung MY](http://www.ncbi.nlm.nih.gov/pubmed/?term=Jung MY%5BAuthor%5D&cauthor=true&cauthor_uid=22515152), [Park SJ](http://www.ncbi.nlm.nih.gov/pubmed/?term=Park SJ%5BAuthor%5D&cauthor=true&cauthor_uid=22515152), [Rijpstra WI](http://www.ncbi.nlm.nih.gov/pubmed/?term=Rijpstra WI%5BAuthor%5D&cauthor=true&cauthor_uid=22515152), [Sinninghe Damsté JS](http://www.ncbi.nlm.nih.gov/pubmed/?term=Sinninghe Damsté JS%5BAuthor%5D&cauthor=true&cauthor_uid=22515152), [Madsen EL](http://www.ncbi.nlm.nih.gov/pubmed/?term=Madsen EL%5BAuthor%5D&cauthor=true&cauthor_uid=22515152)  *et al.*. (2012). Cultivation of a highly enriched ammonia-oxidising archaeon of thaumarchaeotal group I.1b from an agricultural soil. Environ. Microbiol 14(6):1528–1543.

Jung MY, Well R, Min D, Giesemann A, Park SJ, Kim JG *et al*. (2013). Isotopic signatures of N2O produced by ammonia-oxidising archaea from soils. ISME J. 8(5):1115–1125.

Lanfear R, Calcott B, Ho SYW, Guindon S. (2012). Partitionfinder: combined selection of partitioning schemes and substitution models for phylogenetic analyses. Mol Biol Evol 29(6):1695–1701.

[Lebedeva EV](http://www.ncbi.nlm.nih.gov/pubmed/?term=Lebedeva EV%5BAuthor%5D&cauthor=true&cauthor_uid=24278328), [Hatzenpichler R](http://www.ncbi.nlm.nih.gov/pubmed/?term=Hatzenpichler R%5BAuthor%5D&cauthor=true&cauthor_uid=24278328), [Pelletier E](http://www.ncbi.nlm.nih.gov/pubmed/?term=Pelletier E%5BAuthor%5D&cauthor=true&cauthor_uid=24278328), [Schuster N](http://www.ncbi.nlm.nih.gov/pubmed/?term=Schuster N%5BAuthor%5D&cauthor=true&cauthor_uid=24278328), [Hauzmayer S](http://www.ncbi.nlm.nih.gov/pubmed/?term=Hauzmayer S%5BAuthor%5D&cauthor=true&cauthor_uid=24278328), [Bulaev A](http://www.ncbi.nlm.nih.gov/pubmed/?term=Bulaev A%5BAuthor%5D&cauthor=true&cauthor_uid=24278328) *et al.*. (2013). Enrichment and genome sequence of the group I.1a ammonia-oxidising Archaeon “Ca. *Nitrosotenuis uzonensis*” representing a clade globally distributed in thermal habitats. PLoS One 8(11):e80835.

Lehtovirta-Morley LE, Stoecker K, Vilcinskas A, Prosser JI, Nicol GW. (2011). Cultivation of an obligate acidophilic ammonia oxidizer from a nitrifying acid soil. Proc Natl Acad Sci USA 108(38):15892–15897.

Martin DP, Lemey P, Lott M, Moulton V, Posada D, Lefeuvre P. (2010). RDP3: a flexible and fast computer program for analyzing recombination. Bioinformatics 26(19):2462–2463.

Matsutani N, Nakagawa T, Nakamura K, Takahashi R, Yoshihara K, Tokuyama T. (2011). Enrichment of a novel marine ammonia-oxidizing archaea obtained from sand of an eelgrass zone. Microbes Environ 26(1): 23–29.

Mosier AC, Allen EE, Kim M, Ferriera S, Francis CA. (2012a). Genome sequence of “Candidatus *Nitrosoarchaeum limnia*” BG20, a low-salinity ammonia-oxidizing archaeon from the San Francisco Bay estuary. J Bacteriol 194(8):2119–2120.

Mosier AC, Allen EE, Kim M, Ferriera S, Francis CA. (2012b). Genome sequence of “Candidatus *Nitrosopumilus salaria*” BD31, an ammonia-oxidizing archaeon from the San Francisco Bay estuary. J Bacteriol 194(8): 2121–2122.

Ochsenreiter T, Selezi D, Quaiser A, Bonch-Osmolovskaya L, Schleper C. (2003) Diversity and abundance of Crenarchaeota in terrestrial habitats studied by 16S RNA surveys and real time PCR. Environ Microbiol 5(9):787–797.

Paradis E, Claude J, Strimmer K. (2004). APE: Analyses of Phylogenetics and Evolution in R language. Bioinformatics 20(2):289–290.

Park BJ, Park SJ, Yoon DN, Schouten S, Sinninghe Damsté JS, Rhee SK. (2010). Cultivation of autotrophic ammonia-oxidizing archaea from marine sediments in coculture with sulfur-oxidizing bacteria. Appl Environ Microbiol 76(22):7575–7587.

Park SJ, Kim JG, Jung MY, Kim SJ, Cha IT, Kwon K *et al*. (2012b). Draft genome sequence of an ammonia-oxidizing archaeon, “Candidatus *Nitrosopumilus koreensis*” AR1, from marine sediment. J Bacteriol 194(24):6940–6941.

Park SJ, Kim JG, Jung MY, Kim SJ, Cha IT, Ghai R *et al*. (2012b). Draft genome sequence of an ammonia-oxidising archaeon, “Candidatus *Nitrosopumilus sediminis*” AR2, from Svalbard in the Arctic Circle. J Bacteriol 194(24):6948–6949.

[Quince C](http://www.ncbi.nlm.nih.gov/pubmed/?term=Quince C%5BAuthor%5D&cauthor=true&cauthor_uid=21276213), [Lanzen A](http://www.ncbi.nlm.nih.gov/pubmed/?term=Lanzen A%5BAuthor%5D&cauthor=true&cauthor_uid=21276213), [Davenport RJ](http://www.ncbi.nlm.nih.gov/pubmed/?term=Davenport RJ%5BAuthor%5D&cauthor=true&cauthor_uid=21276213), [Turnbaugh PJ](http://www.ncbi.nlm.nih.gov/pubmed/?term=Turnbaugh PJ%5BAuthor%5D&cauthor=true&cauthor_uid=21276213). (2011). Removing noise from pyrosequenced amplicons. [BMC Bioinformatics.](http://www.ncbi.nlm.nih.gov/pubmed/?term=quince+ampliconnnoise) 12:38.

Santoro AE, Casciotti KL. (2011). Enrichment and characterization of ammonia-oxidizing archaea from the open ocean: phylogeny, physiology and stable isotope fractionation. ISME J 5(11):1796–1808.

Spang A, [Poehlein A](http://www.ncbi.nlm.nih.gov/pubmed/?term=Poehlein A%5BAuthor%5D&cauthor=true&cauthor_uid=23057602), [Offre P](http://www.ncbi.nlm.nih.gov/pubmed/?term=Offre P%5BAuthor%5D&cauthor=true&cauthor_uid=23057602), [Zumbrägel S](http://www.ncbi.nlm.nih.gov/pubmed/?term=Zumbrägel S%5BAuthor%5D&cauthor=true&cauthor_uid=23057602), [Haider S](http://www.ncbi.nlm.nih.gov/pubmed/?term=Haider S%5BAuthor%5D&cauthor=true&cauthor_uid=23057602), [Rychlik N](http://www.ncbi.nlm.nih.gov/pubmed/?term=Rychlik N%5BAuthor%5D&cauthor=true&cauthor_uid=23057602) *et al.*. (2012). The genome of the ammonia-oxidizing Candidatus *Nitrososphaera gargensis*: insights into metabolic versatility and environmental adaptations. Environ Microbiol 14(12):3122–3145.

Tamura K, Stecher G, Peterson D, Filipski A, Kumar S. (2013). MEGA6: Molecular Evolutionary Genetics Analysis version 6.0. Mol Biol Evol 30(12):2725–2729.

[Tourna M](http://www.ncbi.nlm.nih.gov/pubmed/?term=Tourna M%5BAuthor%5D&cauthor=true&cauthor_uid=18325029), [Freitag TE](http://www.ncbi.nlm.nih.gov/pubmed/?term=Freitag TE%5BAuthor%5D&cauthor=true&cauthor_uid=18325029), [Nicol GW](http://www.ncbi.nlm.nih.gov/pubmed/?term=Nicol GW%5BAuthor%5D&cauthor=true&cauthor_uid=18325029), [Prosser JI](http://www.ncbi.nlm.nih.gov/pubmed/?term=Prosser JI%5BAuthor%5D&cauthor=true&cauthor_uid=18325029). (2008). Growth, activity and temperature responses of ammonia-oxidizing archaea and bacteria in soil microcosms. Environ Microbiol 10(5):1357-64.

Tourna M, [Stieglmeier M](http://www.ncbi.nlm.nih.gov/pubmed/?term=Stieglmeier M%5BAuthor%5D&cauthor=true&cauthor_uid=21525411), [Spang A](http://www.ncbi.nlm.nih.gov/pubmed/?term=Spang A%5BAuthor%5D&cauthor=true&cauthor_uid=21525411), [Könneke M](http://www.ncbi.nlm.nih.gov/pubmed/?term=Könneke M%5BAuthor%5D&cauthor=true&cauthor_uid=21525411), [Schintlmeister A](http://www.ncbi.nlm.nih.gov/pubmed/?term=Schintlmeister A%5BAuthor%5D&cauthor=true&cauthor_uid=21525411), [Urich T](http://www.ncbi.nlm.nih.gov/pubmed/?term=Urich T%5BAuthor%5D&cauthor=true&cauthor_uid=21525411) *et al.*. (2011). *Nitrososphaera viennensis*, an ammonia oxidising archaeon from soil. Proc Natl Acad Sci U S A 108(20):8420–8425.

Treusch AH, Leininger S, Kletzin A, Schuster SC, Klenk H-P, Schleper C. (2005). Novel genes for nitrite reductase and Amo-related proteins indicate a role of uncultivated mesophilic crenarchaeota in nitrogen cycling. Environ Microbiol 7(12):1985–1995.

Xia X. (2013). DAMBE5: a comprehensive software package for data analysis in molecular biology and evolution. [Mol Biol Evol](http://www.ncbi.nlm.nih.gov/pubmed/?term=xia+2013+dambe) 30(7):1720-1728.
